# Advances and Challenges on Management of Gastrointestinal Stromal Tumors

**DOI:** 10.3389/fonc.2018.00135

**Published:** 2018-05-07

**Authors:** Lin Mei, Wei Du, Michael Idowu, Margaret von Mehren, Sosipatros A. Boikos

**Affiliations:** ^1^Hematology, Oncology and Palliative Care, Massey Cancer Center, Virginia Commonwealth University, Richmond, VA, United States; ^2^Department of Pathology, School of Medicine, Virginia Commonwealth University, Richmond, VA, United States; ^3^Fox Chase Cancer Center, Philadelphia, PA, United States

**Keywords:** GIST, imatinib, *KIT*, *PDGFRA*, *SDH*, *NF1*, *SDHCme*

## Abstract

Gastrointestinal stromal tumors (GISTs) originate from interstitial cells of Cajal and account for over 5,000 newly diagnosed cases in the United States. The discovery of activated *KIT* and *PDGFRA* mutations and introduction of imatinib revolutionized the treatment strategy and opened up the new era of target therapy for solid tumors. Although surgery remains the primary modality of treatment for curative purpose, almost half of the patients experienced disease recurrence. Tailoring (neo)-adjuvant treatment with imatinib is ongoing to meet the need for an effective therapy. Currently, two drugs (sunitinib and regorafenib) have obtained Food and Drug Administration approval for GISTs after imatinib failure. However, most of the patients eventually progress due to primary or secondary resistance. Deeper understanding of the molecular mechanisms will guide us to develop personalized strategies in the future. Discussion in this review includes current standard management and the most recent advances and multiple ongoing clinical trials with different approaches. This review will provide further steps to be taken to conquer refractory disease.

## Introduction

Gastrointestinal stromal tumors (GISTs) are known as the most common mesenchymal tumor of gastrointestinal (GI) system in the United States (1) with the incidence of 10–15 cases per million (2). Median age of diagnosis is around the mid-50s (3). Historically, unless it was completely resected, GIST was a devastating disease due to poor response to chemotherapy and radiotherapy (4). Smooth muscle cells were considered to be the cell type of origin, given the spindle cell morphology. A key finding for GIST classification was clarified due to its similarity to the interstitial cell of Cajal, a stromal cell that serves as the pacemaker for GI tract (5). In 1998, the discovery of activated *KIT* (CD117) mutation significantly reshaped the biological understanding (6) as well as the subsequently identified mutations in platelet derived growth factor receptor α (PDGFRA) (7). Mutations in both receptors drive downstream intracellular pathways and lead to tumorigenesis. Immunohistochemistry study of DOG1 is particularly helpful in diagnosing GISTs that do not express KIT (8, 9). The imatinib, a tyrosine kinase inhibitor (TKI), was originally evaluated in clinical trials for BCR/ABL positive chronic myelogenous leukemia with great success. The homologous structure between KIT, PDGFRA, and ABL kinases facilitated the introduction of imatinib to the therapy of GISTs in 2000 (10, 11) and the Food and Drug Administration (FDA) granted the approval in 2002. Nowadays, there is no doubt that the treatment for GIST set up a paradigm for use of targeted therapy in solid tumors. Approximately 85–90% of GISTs harboring *KIT* or *PDGFRA* mutations benefit from imatinib treatment before or after surgery and in the setting of unresectable/metastatic disease (12, 13), except specific mutation such as PDGFRA exon 18 D842V (14). The remaining 10–15% of GISTs without *KIT* or *PDGFRA* mutations are classified as wild-type (WT) GIST. They lack response to imatinib. In this group, several mutations have been identified including succinate dehydrogenase (SDH) complex subunits, neurofibromatosis type 1, *BRAF*, and other genes (15). These GISTs remain a great therapeutic challenge still. Further trials led to two more drugs approval by the FDA, sunitinib and regorafenib, which provide options after failure of imatinib. This study reviews the role of targeted therapies in non-metastatic and metastatic GIST, as well as future direction and ongoing clinical trials.

## Risk Factors of Resectable GISTs

Macroscopic complete surgical resection (R0/R1) remains the major curative approach for GISTs. Any tumor more than 2 cm, symptomatic disease (e.g., bleeding and obstruction) or a tumor that is progressively increase in size should be considered for resection (16). For early stage disease, wedge resection with 1–2 cm margin or segmental resection is usually sufficient because primary GISTs generally displace rather than invade adjacent tissue (17). The risk of lymph node involvement is low, unless the tumor is SDH-deficient. Microscopic negative margins (R0 resection) is the goal of resection, though R1 resection with microscopic positive margins do not usually require re-excision (18). On the basis of American College of Surgeons Oncology Group (ACOSOG) Z9000 and Z9001 clinical trials, there was no difference in recurrence free survival (RFS) for patients undergoing R0 or R1 resection, regardless of adjuvant treatment (19, 20). Only tumor rupture, tumor size, mitotic index, and location are associated with increased risk of recurrence, which have been suggested by National Institutes of Health (NIH) consensus (21) and Miettinen’s classification system (16). This finding may indicate the primary prognostic factor is the inherent biological feature rather than resection status.

In the pre-imatinib era, the estimated 15-year RFS after surgery was 60% for all stages of operable GIST (22). An increased risk for recurrence was associated with an increased mitotic rate [>5/50 high power field (HPF)], large tumor size (>10 cm), and location (small bowel) (23). Further population-based cohort studies added tumor rupture as a new adverse prognostic factor (22). In addition, advances in surgical techniques brought better outcomes and shorter hospital stays. Novitsky et al. (24) and Otani et al. (25) reported the safety and low morbidity of utilizing laparoscopic resection for gastric GISTs (1–8.5 and 2–5 cm, respectively) with 100% negative resection margins compared with open resection. 3 years follow-up showed >90% patients remained disease free.

To date, although more and more gene mutations have been identified in GIST, none of them are incorporated into predictive models. Some evidence suggests that mitotic rate may be less predictive of biologic behavior in SDH-deficient GISTs (26) and depletions affecting codons 557–558 at KIT exon 11 have a greater risk of recurrence than other exon mutations (2, 27).

## Neoadjuvant Treatment for Resectable GISTs

To downstage large advanced GISTs or achieve R0/R1 resection of poorly positioned tumors, neoadjuvant treatment, particularly imatinib, is considered before surgery. In 2006, first case report of using imatinib in the neoadjuvant setting obtained complete pathological response of a pelvic GIST patient (28). Subsequently, the Radiation Therapy Oncology Group 0132 trial was the first prospective phase II trial to evaluate the efficacy of imatinib before borderline resectable tumor (29). Thirty patients with primary GIST and twenty-two with recurrent GIST were administered imatinib 600 mg daily for 8–12 weeks preoperatively and extended for 2 years after surgery. Preoperative imatinib was well tolerated and did not affect surgical outcomes. The response rate at the time of surgery using RECIST criteria was 4.5 and 7% in recurrent and primary disease, respectively. Eventually 77% of the patients achieved R0/R1 resection. RFS at 2 years was 82.6%, and 2 years overall survival (OS) was 93% (29). Neoadjuvant treatment provides a chance to avoid morbid procedure. However, it is unclear whether the gain of RFS was attributing to 2 years adjuvant imatinib or not. In this study, the response rate to imatinib is low. Partly it is because of the limited duration of preoperative imatinib. In addition, RECIST criteria for evaluation of treatment response underestimate imatinib-induced cytoreduction (30). Although preoperative imatinib is useful to reduce tumor size, and there is no definitive evidence that it leads to increased OS. Long-term follow-up results from the same study showed 5 years RFS of 56% and 5 years OS of 77%. The prognosis was not correlated with surgical resection status (31). A phase II prospective APPOLLON trial evaluated overall tumor response in 41 patients with locally advanced *KIT*- and *PDGFRA*-positive GISTs. Imatinib 400 mg was taken daily for 6 months before the surgery. R0 resection was obtained 30/34 (88.2%) of patients; progression free survival (PFS) at 3 years was 85.2% (32); and no OS data were reported.

Newly published phase II study data from Asia further illustrate the strategy of utilizing neoadjuvant treatment. Specifically for large gastric GISTs (≥10 cm), patients received neoadjuvant imatinib (400 mg/day) for 6–9 months. In 53 patients who received neoadjuvant therapy, 3 patients refused surgery and 4 patients withdrew from the trial. 46 patients completed ≥6 months treatment, with response rate of 62% and R0 resection rate was 91%. 2-year OS was 89%. This study validated that the minimum duration of 6 months neoadjuvant imatinib therapy was required. Preoperative treatment was beneficial for patients who have large tumors (median size in this study was 12 cm) to achieve R0 resection and 94% of the patients avoided total gastrectomy. Most importantly, in this study, genotyping was carried out before neoadjuvant imatinib. One patient did not start neoadjuvant imatinib due to *PDGFRA* exon 18 D842V mutation. This study demonstrates that *KIT* exon 11 mutation tumors had high R0 resection rate post neoadjuvant imatinib. In addition, two patients with WT-GIST had successful R0 resection as well (33). This study demonstrated that mutational status testing is important to determine the potential benefit of neoadjuvant imatinib treatment. However, due to it is only a single-arm study and limited follow-up period, further studies are warranted to evaluate the survival benefit of preoperative treatment.

## Adjuvant Treatment for Resectable GISTs

Historically, as many as 50% of patients died of recurrent disease despite complete resection of primary tumor with recurrence associated with tumor size (34). Therefore, given the known benefit of imatinib, the question of whether adjuvant imatinib would improve survival postoperatively was tested. The ACOSOG Z9000 Intergroup phase 2 trial was the first prospective study showing that 1 year of adjuvant imatinib did prolong RFS after complete resection in high-risk population compared with historical controls. RFS at 1, 3, and 5 years is 96, 60, and 40%, respectively (19). Subsequently, the ACOSOG Z9001 phase 3 trial clearly demonstrated that an increase RFS among patients with 1 year adjuvant imatinib after resection of 3 cm or larger tumors compared with placebo. Other risk features included mitoses greater than 5 per 50 HPF. The 1 year RFS was 98% with imatinib versus 83% with placebo, a statistically significant difference; and no survival improvement was observed (20, 35).

The next question that was tested was whether 1 year of adjuvant treatment is sufficient. In 2015, European Organization for Research and Treatment of Cancer (EORTC) 62024 trial reported on 2 years of imatinib 400 mg daily or observation only in patients with high- or intermediate-risk GISTs after R0/R1 surgery. Imatinib failure-free survival (IFFS) was a novel surrogate endpoint proposed in this trial, to avoid many years follow-up to determine the OS benefit. IFFS was not statistically different in the imatinib arm compared with the observation arm (87 versus 84%), as well as 5-year OS (100 versus 99%). RFS was significantly higher at 3 years (84 versus 66%) and 5 years (69 versus 63%) in the imatinib arm (36). For the intermediate-risk according to current classification, there was no improvement of IFFS. Half of the patients had non-gastric GISTs and mutational data were not available.

The Scandinavian/German SSG XVIII/AIO trial, a randomized, open-label phase 3 study, compared 1 year versus 3 years of postoperative imatinib with 400 mg daily after R0/R1 resection of high-risk primary or metastatic GIST (37). At a median follow-up of 4.5 years, both RFS and OS favored 3 years of therapy. The 5-year RFS in 3 years and 1 year treatment groups were 65 and 48%, respectively. 5-year survival was significantly longer in patients assigned to 3 years of imatinib (92 versus 82%, *P* = 0.02). A *KIT* or *PDGFRA* mutation was detected in 91% of tumors. However, tumors with *KIT* exon 9 mutation or WT-GISTs did not have significant clinical benefit (37). The result from EORTC and SSG trials further suggested the adjuvant imatinib treatment should be carefully applied to high-risk patients and genotype should also be taken into consideration. For example, in the advanced/metastatic setting, the *PDGFRA* exon 18 D842V mutated GISTs have no benefit from imatinib (14) and higher-dose of imatinib (800 mg daily) is recommended by some institutions for *KIT* exon 9-mutated GIST (38). Biologically, the results may be extrapolated to adjuvant treatment. In addition, in spite of frequent recommendation of 3-year adjuvant imatinib therapy based on SSG trial, both ACOSOG Z9001 and EORTC trials failed to show survival improvement (35, 36). The OS benefit was confirmed by Joensuu et al. (39) in the second planned analysis of the SSGXVIII/AIO trial after a median follow-up of 90 months. The 3-year group demonstrated improved RFS (71 versus 52%, *P* < 0.001) and survival benefit (92 versus 85%, *P* = 0.036). The most beneficial mutational subgroup was *KIT* exon 11 (39). Most recently, the same group published the final genotypic analysis data. Of the 341 patients from SSG XVIII/AIO trial, the study again found that *KIT* exon 11 deletion or insertion-deletion mutations involved codons 557 and/or 558 had better RFS following 3 years adjuvant imatinib treatment compared with 1 year, but not there was not a statistical difference in the in exon 11 substitution mutations, exon 9 mutations, *PDGFRA* mutation (including D842V mutation), or WT-GISTs; there were too few cases with other mutations to make conclusions (40). This pivotal study further elucidated 3-year adjuvant imatinib treatment helped the high recurrent risk group (> 10 mitoses/50 HPFs and *KIT* exon 11 mutation) the most (40). However, prolongation of treatment for more than 3 years is controversial and there is currently a lack of evidence. The NCCN guidelines currently recommend at least 3 years treatment. Two ongoing randomized trials (NCT02413736 and NCT02260505) are currently comparing 3 versus 5 or 6 years of adjuvant imatinib treatment would provide further benefits.

For low risk GISTs management, observation is still the standard of care after R0/R1 resection. If a patient underwent neoadjuvant imatinib treatment, it is recommended to continue it after surgery to accomplish cumulative 3-year course. The rationale for continuation of imatinib is because the mitotic count or biological markers from the postoperative samples is no longer reliable for assessing recurrence risk accurately. In patients who progress on the standard dose of imatinib (400 mg/day), dose escalation to 800 mg/day may be considered (17).

## Treatment for Resectable WT-GISTs

WT-GIST was initially defined as the absence of both *KIT* and *PDGFRA* mutations. The unique feature of this subtype of GIST is it has poor response to TKIs, including imatinib (12). Recent studies have identified additional genetic mutations in this particular group that prompt us to revisit so-called WT-GISTs. Based upon the molecular features, a new classification for a subset of these tumors based upon the presence or absence of SDH activity, namely SDH-competent or SDH-deficient subgroups.

Due to the rarity of WT-GISTs, there has been a lack of definitive recommendations for this entity. The limited experience from subgroup analysis of ACOSOG Z9001 (32 WT-GIST patients) (20) and SSG XVIII/AIO trial (19 WT-GIST patients) (37) did not detect any benefit from postoperative imatinib treatment. A recent report from the NIH pediatric and WT-GIST clinic added valuable information to the overall picture (41). The WT-GIST clinic was established in 2008. It included patients who had undergone surgical resection of their tumor. With a median follow-up of 4.1 years, 5- and 10-year EFS in the patients seen at that clinic was 24 and 16%, respectively, showing a more indolent process than *KIT/PDGFRA*-mutated GISTs. The majority of lesions were located in the stomach (83%). The prognosis was related to mitotic index (>5 mitoses/50 HPF) and metastatic status; R0 resection, SDH mutation, or anatomic location were not prognostic. In the setting of hemorrhage, perforation, pain or obstruction, surgery was still the cornerstone of management (41). Although it provides the most comprehensive cohort study for WT-GISTs, the role of adjuvant TKI treatment was not reported in this study. Follow-up with this cohort regarding the evolution of targeted therapy will further help our understanding of adjuvant treatment in WT-GISTs (41).

## Target Therapy for Advanced or Metastatic GISTs

### Imatinib

Traditionally, GISTs were believed to be chemotherapy and radiotherapy resistant. Surgical resection was the only effective treatment option available before 2000. Median survival was about 10–20 months for unresectable or metastatic disease (42). In 1998, the breakthrough finding of activated *KIT* was a crucial diagnostic marker as well as potential therapeutic target for GIST, opening a new era for GIST therapy (6). Only 2 years later, imatinib was tested due to its potent antagonism of *KIT* in an *in vitro* cellular model (10). Subsequently a patient with metastatic GIST was treated with imatinib and demonstrated a significant response (11), further affirming targeting aberrant tyrosine kinase signaling can be therapeutic. This favorable outcome from a case report triggered the subsequent clinical trials. Demetri et al. reported total 147 patient cohort study randomized to receive imatinib 400 or 600 mg daily. The overall response rate was 54% and there was good tolerability (43). Long-term results from the same study validated identical efficacy of 400 and 600 mg daily dose. Nearly 50% of the patient with advanced GIST survived for more than 5 years with ORR of 68% and PFS of 24 months (44). The highest feasible dose of imatinib was identified as total 800 mg daily by the EORTC phase I and phase II studies (45, 46). Based on the successful outcome from the early phase clinical trials, two multi-center phase III studies tested two different daily doses, 400 mg daily versus 400 mg twice daily. The EORTC study recruited 946 patients and demonstrated that the 400 mg/day had a similar response rate compared with 800 mg/day. The high-dose imatinib treatment did lead to a significantly longer PFS at the expense of higher treatment interruption (64 versus 40%) or dose reductions (60 versus 16%) (47). The Southwest Oncology Group S0033 trial was conducted in 148 centers across United States and Canada enrolled 746 patients with metastatic or surgically unresectable GIST. Median OS was nearly 5 years (55 versus 51 months) in the 400 mg/daily and 800 mg/daily groups, respectively, and was not statistically different between the arms. Likewise, neither ORR nor PFS revealed any differences. As expected, the high-dose group had more grade 3–5 toxicities (63 versus 43%). Therefore, the study concluded that high-dose does not provide clinical advantage over standard dose (48). The meta-analysis evaluating these two randomized trials concluded the same result (38). Recently, 10-year follow-up results were updated from the EORTC international study. The median PFS was 1.7 and 2.0 years (*P* = 0.18) in the 400 and 800 mg arms, and median survival was 3.9 years in both arms. Only 10% of patients were progression free at 10 years. With longer follow-up, there is a lack of data to support a difference between the two dose levels (49).

Though mutational analysis was not mandatory to enroll on the protocols, subset of tumors was genotyped. *KIT* exon 9-mutated GIST was shown to benefit from high-dose imatinib for both PFS and OS, while WT-GIST more favored standard dose (49). Based on these data, imatinib 400 mg/daily is the standard of care; however, higher-dose (800 mg/daily) is a consideration for patients that have progressed on 400 mg/daily or for tumors that harbor *KIT* exon 9 mutations. In addition, *PDGFRA* D842V mutated tumors were resistant to therapy (50). With the greater insight of molecular profiles, we have better understanding of the potential for response to imatinib. Thus, the NCCN guideline strongly recommends mutation testing or genotyping for *KIT* and *PDGFRA*.

How long imatinib should be given if the disease is controlled was a question explored by the French Sarcoma Group. BFR14, a phase 3 trial, explored whether imatinib can be interrupted beyond 1 year of treatment in patients with advanced or unresectable disease. A considerably higher rate of disease progression was reported in interrupted group (81 versus 31%) without impairment of quality-of-life (51). The same group then tested interruption at 3 years. Similarly, after a median follow-up of 35 months, the 2-year PFS was 80 versus 16% in continuation group and interruption group, respectively (52). Likewise, cognate result was observed in 5 years interruption group (53). These data lead to the conclusion that ongoing imatinib maintenance is crucial for advanced/metastatic GISTs until there is evidence of disease progression or intolerance (51).

When patients progress after treatment with approved TKIs, including sunitinib, there are few treatment options left. A phase 3 RIGHT trial, tried to address this problem by reintroducing imatinib. Forty-one patients were assigned to rechallenge imatinib versus placebo after progression on sunitinib. PFS was 1.8 months with imatinib compared with 0.9 month (*P* = 0.005) with placebo (54). BFR14 trial also recapitulated the response of rechallenge imatinib if demonstrated progressive disease after discontinuation of imatinib (53). It provides a new strategy by continuous kinase inhibition by rechallenge with imatinib as an effective therapeutic approach if new investigational drugs are not readily available.

### Sunitinib

The majority of patients develop resistance to treatment with imatinib, either due to primary or secondary resistance. Approximately 10% of patients with GISTs have primary resistance (progression within the first 6 months of starting imatinib) primarily because of the tumor mutational status (13). Secondary resistance is defined as disease progression after initial response to imatinib, largely due to acquired mutation in *KIT* or *PDGFA*. Therefore, there is a need for additional treatments with potent activity against *KIT* and *PDGFRA*. Sunitinib is approved worldwide for metastatic GISTs in patients with imatinib resistance or intolerance (55). In the pivotal phase 3 trial, 312 patients were enrolled to receive sunitinib or placebo after failure of imatinib with the dose of 50 mg daily, 4 weeks on and 2 weeks off. In spite of a very low response rate (only 7%), sunitinib demonstrated prolonged PFS (6.3 versus 1.5 months) and a fourfold higher TTP (27 versus 6 weeks), which were the designed primary endpoints. Numerically, OS was also better though it was not significant, as the study was unblinded and all patients on the placebo arm were crossed over to active therapy following the first interim analysis (56). To achieve better efficacy, the dosing of 37.5 mg sunitinib daily without interruption was also tested in an open-label phase 2 trial with similar outcomes. The response rate was about 13% and PFS was 34 weeks (57).

Although sunitinib has a wider spectrum of kinase inhibition, it is overall well tolerated. The most frequent adverse events are fatigue, diarrhea, hand-foot syndrome, or hypothyroidism, which can be managed by dose modification or interruption (56, 57). In a geriatric population, a report has suggested it might have a negative impact on cognitive function (58). Hypertension induced by sunitinib, associated with improved clinical outcomes, had a low incidence and was manageable (59).

Progression free survival and OS were significantly higher in *KIT* exon 9 mutation and WT-GIST subtypes with sunitinib, as well as *KIT* exon 11 mutations with secondary *KIT* exon 13 or 14 mutations (14), while secondary KIT exon 17 and 18 mutations involving the *KIT* activation loop had poor outcomes with sunitinib (60). 18F-fluorodeoxyglucose positron emission tomography was assessed as a predictive tool to individualize patient with sunitinib therapy in 4 weeks (61). Recently, a large real world study (Study 1036; NCT00094029) in which 1,124 sunitinib-treated patients were evaluated was reported. A significantly better PFS (median was 7.1 months) was observed in KIT exon 9 mutation compared with exon 11 mutation (hazard ratio = 0.59). Longer OS and ORR were reported as well (62). Combined with the existing evidence, sunitinib offered effectiveness as a post-imatinib therapy, regardless of mutational status.

### Regorafenib

Regorafenib is another oral multi-targeted TKI with activity on oncogenic pathways (KIT, RET, PDGFR, FGFR, and BRAF) and angiogenesis pathways (VEGF1-3 and TIE2) (4). It has been approved by FDA for GIST patients previously treated with imatinib and sunitinib, as well as colorectal cancer and hepatocellular cancer. In 2011, Wilhem et al. first reported regorafenib suppressed growth of GIST *in vitro* as well as xenograft mouse model (63). Then a phase II study for treating GIST after failure of imatinib and sunitinib was reported in 2012, demonstrating that regorafenib at a dose of 160 mg daily for 3 weeks in a 4 weeks cycle had clinical benefit rate of 79% with median PFS of 10 months (64). On the basis of these promising data, a phase 3 trial (GRID trial) was performed in 199 patients who had progressed on previous imatinib and sunitinib therapy (65). Disease control rate was dramatically improved in regorafenib arm compared with placebo (52 versus 9%). Median PFS was 4.8 versus 0.9 months favoring regorafenib. No difference was observed in OS due to the crossover design. Grade III or higher toxicity was present in about 20% of patients (65). This evidence lead to FDA accelerated approval of regorafenib as the third-line agent in 2013.

## Other New Target Therapies

Despite success of imatinb, sunitinib and regorafenib, eventually most patients develop resistant to these therapies, mainly due to acquired mutations. Several other TKIs have been evaluated in this setting, however, none of them have led to FDA approval to date.

Sorafenib, structurally closely related to regorafenib, targets multiple tyrosine kinases including KIT and PDGFRA. Currently it is approved for metastatic hepatocellular carcinoma, renal cell carcinoma, and differentiated thyroid cancer. A single-arm phase 2 trial had reported with 31 patients with GIST who failed both imatinib and sunitinib. The response rate was 13%. Median PFS and OS were 4.9 and 9.7 months (66).

Nilotinib is a second-generation TKI derived from imatinib. It has potency similar to that of imatinib against KIT and PDGFRA. *In vitro* activity of nilotinib suggested greater inhibitory effect against BCR-ABL and comparable KIT/PDGFRA effect (67). ENESTnd trial established its role in frontline therapy of newly diagnosed chronic myeloid leukemia with better efficacy than imatinib (68). Therefore, ENESTg1 trial was designed as a randomized phase 3 trial to assess the efficacy and safety of nilotinib versus imatinib as first-line therapy for patients with advanced GISTs (69). From 2009 to 2011, 647 patients were enrolled. The 2 years PFS was higher in the imatinib group than nilotinib group (59 versus 51%), primarily due to poorer disease control by nilotinib in the group with *KIT* exon 9-mutated GIST. In this regard, this trial was terminated early as the futility boundary was crossed at the interim analysis (69). In the third-line setting, nilotinib also failed to demonstrate significant activity in patients with prior imatinib and sunitinib treatment (70), though the best supportive care (BSC) group was allowed to continue imatinib or sunitinib. For the moment, nilotinib is not recommended for broad use for GIST. Nilotinib has adverse effect for *KIT* exon 9-mutated GIST and should be avoided. Future studies might identify some patient subsets might of clinical benefit.

Pazopanib, a multi-targeted angiogenesis inhibitor, has shown activity against non-GIST soft-tissue sarcoma in the PLAETTE trial (71) and achieved a favorable quality-of-life profile compared to sunitinib for metastatic renal cell carcinoma (72). A phase 2 PAZOGIST trial was performed to assess the activity of pazopanib in imatinib and sunitinib-resistant GIST or refractory to other therapies (73). The primary endpoint, 4-month PFS was higher in the pazopanib group than the BSC alone group (45 versus 17%). Median PFS was 3.4 months in pazopanib group and 2.3 months in BSC. OS was not significant (HR = 0.94) (73). Nevertheless, regorafenib which is approved for third-line therapy has median PFS of 4.8 months (65). Another phase 2 study of pazopanib conducted in USA did not show such high anti-tumor activity, with median PFS of only 1.9 months, though median lines of treatment was 3 (74). Accordingly, because of the low proportion of patients obtaining a response and limited evidence, pazopanib should be used in selected patients with GIST or those with no clinical trial options following progression with standard therapies. Interestingly, the phase 2 study suggested that the drug may be of benefit for SDH-deficient WT-GIST (74).

Masitinib is another highly selective TKI with inhibitory effect of KIT as well as WT-GIST in a phase 1 study (75). A pilot phase 2 study compared masitinib with imatinib in treatment-naive metastatic GIST patients (76). There was a similar safety and response profile to imatinib, with ORR of 53%, disease control rate of 97% and mPFS was 41.3% (76). Another phase 2 trial further evaluated masitinib in the second-line after failure of imatinib, using sunitinib as a comparative control. The mOS was significantly longer for patients receiving masitinib (HR = 0.27) with a 12.4 months survival advantage. Patients experienced less toxicity from masitinib than those receiving sunitinib (52 versus 91%) (77). This encouraging result is awaiting to be validated from an ongoing phase 3 trial (NCT01694277).

To date, drugs targeting KIT and PDGFRA have revolutionized GIST treatment. However, resistance to existing drugs and disease progression are not uncommon within a few years of treatment. Significant effort has been applied to find alternate agents with other mechanisms of action or combination therapy to circumvent the resistance without adding toxicity. Several studies with novel agents, such as BLU-285, crenolanib in patient harboring highly resistant mutation of PDGFRA D842V (78, 79), dabrafenib in BRAF-mutated GIST (80), are being tested with some early signs of benefit. ETV1 has emerged as a highly specific target in treatment of GISTs. Notably, MEK inhibitor had synergistic effect with imatinib (81). A phase 1 clinical trial (NCT01991379) is ongoing highlighting the rapid translation from bench work to bedside. Immunotherapy, which has revolutionized our concept of anti-tumor treatment in other tumor types, also maybe a new avenue for treating GISTs. Pre-clinical studies indicate that program cell death protein 1 (PD-1) signaling is correlated with clinical outcome and imatinib treatment (82). In a single-arm phase 2 study, Toulmonde et al. investigated the efficacy of pembrolizumab combined with metronomic cyclophosphamide in sarcoma including 10 GIST patients. The 6-month non-progression rate was observed in only 11.1% GIST patients (83). Hereby, due to the disappointing result, further strategies are warranted to assess the combination of anti-PD-1 with other approaches targeting tumor immune microenvironment. Current ongoing clinical trials in clinicaltrial.gov have been summarized in Tables 1 and 2.

**Table 1:** Selected clinical trials.

Identifier	Intervention	Title	Target	Design	Purpose
NCT02365441	Regorafenib	Imatinib alternating with regorafenib for advanced gastrointestinal stromal tumor (GIST)	General	Phase II/first-line	Treatment
NCT02638766	Regorafenib	Regorafenib in metastatic and/or unresectable KIT/PDGFR wild-type GIST	General	Phase II/first-line	Treatment
NCT02606097	Regorafenib	Regorafenib in GIST with secondary c-KIT Exon 17 mutation	c-KIT	Phase II/second-line	Treatment
NCT02889328	Regorafenib	Continuous versus intermittent dosing of regorafenib in GIST	General	Phase II/second-line	Treatment
NCT02164240	Sunitinib	Sunitinib alternating with regorafenib in metastatic/unresectable GIST (SURE)	General	Phase Ib/second-line	Treatment
NCT01396148	Sunitinib	Sunitinib in young patients with advanced GIST (non-mutant c-KIT)	General	Phase I/II/first-line	Treatment
NCT00700258	Sunitinib	Registry for sunitinib in GIST (STAR-TOR)	General	Cohort	Observational
NCT01541709	Imatinib	Imatinib 800 mg in metastatic/unresectable GIST harboring KIT Exon 9 mutation	c-KIT	Phase II/first-line	Treatment
NCT02576080	Imatinib	Efficacy of imatinib with intermediate/high-risk genomic grade GIST	General	Phase III	Diagnostic
NCT02413736	Imatinib	3 versus 5 years of adjuvant imatinib with operable GIST	General	Phase III	Treatment
NCT02216578	Cabozantinib	Ph II CABOGIST in metastatic GIST	General	Phase II/second-line	Treatment
NCT02847429	Crenolanib	Crenolanib in subjects with platelet derived growth factor receptor α (PDGFRA) D842V mutated GIST	PDGFRA	Phase III/first-line	Treatment
NCT02342600	Pazopanib + trametinib	Trametinib and pazopanib in metastatic/recurrent GIST (SARC029)	General	Phase II/second-line	Treatment
NCT02776878	Dasatinib	Efficacy and safety of dasatinib in refractory metastatic GIST	General	Phase Ib/II/second-line	Treatment
NCT02034110	Dabrafenib + trametinib	Efficacy and safety of dabrafenib and trametinib in BRAF V600E-mutated rare cancers	BRAF	Phase II	Treatment
NCT01991379	MEK162 (MEK inhibitor)	MEK162 in combination with imatinib untreated advanced GIST	ETV	Phase Ib/II/first-line	Treatment
NCT02607332	Paclitaxel	Paclitaxel in advanced GIST after failure to imatinib and sunitinib	General	Phase II/second-line	Treatment
NCT01738139	Ipilimumab + imatinib	Ipilimumab and imatinib in advanced cancer	Program cell death protein 1 (PD-1)	Phase I/first-line	Treatment
NCT02880020	Ipilimumab	Nivolumab with or without ipilimumab in metastatic GIST	PD-1/CTLA-4	Phase II/second-line	Treatment
NCT02982486	Nivolumab + ipilimumab	Immune therapy in non-resectable sarcoma with deficient MMR	PD-1/CTLA-4	Phase II/first-line	Treatment
NCT02406781	Pembrolizumab +cyclophosphamide	Combination of pembrolizumab and metronomic cyclophosphamide in patient with advanced sarcomas (PEMBROSARC)	PD-1	Phase II/first-line	Treatment
NCT02686944	Intuvax (cancer Vaccine)	Safety of intuvax administered intra-tumorally in patient with GIST	Immune therapy	Phase I/second-line	Treatment
NCT01389583	AUY922 (HSP inhibitor)	A study of AUY922 for GIST	HSP	Phase II/second-line	Treatment
NCT02257541	BGJ398 (FGFR inhibitor)	BGJ398 in combination with imatinib in patient with advanced GIST	FGFR	Phase I/b/II/second-line	Treatment
NCT02508532	BLU-285 (PDGFRA D842V mutant inhibitor)	BLU-285 in patient with GISTs	PDGFRA	Phase I/second-line	Treatment
NCT01907607	Palbociclib (CDK4/6 inhibitor)	Efficacy and safety of PD-0332991 (palbociclib) in advanced GIST refractory to imatinib and sunitinib (CYCLIGIST)	CDK4/6	Phase II/second-line	Treatment
NCT02401815	PLX9486 (c-KIT inhibitor)	PLX9486 with or without PLX3397 in patient with advanced solid tumors	c-KIT	Phase Ib/second-line	Treatment
NCT02452424	PLX3397 (CSF-1 inhibitor) + pembrolizumab	Combination study of PLX3397 and pembrolizumab to treat advanced melanoma and other solid tumors	CSF-1/PD-1	Phase I/IIa/second-line	Treatment
NCT02232620	BBI503 (multi-kinase inhibitor)	BBI503 in adult patients with advanced GIST	General	Phase II/second-line	Treatment
NCT02571036	DCC-2618 (C-KIT inhibitor)	Safety, tolerability and PK study of DCC-2618 in advanced GIST	c-KIT	Phase I/second-line	Treatment
NCT02071862	CB-839 (Glutaminase inhibitor)	Glutaminase inhibitor CB-839 in solid tumor [succinate dehydrogenase (SDH)-deficient GIST]	SDH	Phase I/second-line	Treatment

**Table 2:** Genotype specific gastrointestinal stromal tumor treatment options.

Genetic/epigenetic alteration	Exon	Imatinib treatment	Other selected treatment choices
*KIT*	Exon 9	400 mg BID	Sunitinib
	Exon 11	400 mg QD	Regorafenib
			DCC-2618 trial (NCT02571036)
*PDGFRA*	Exon 12	400 mg QD	BLU-285 (NCT02508532)
	Exon 14	400 mg QD	
	Exon 18 (D842V)	Imatinib resistant	Dasatinib, Crenolanib trial (NCT028474729)
			DCC-2618 trial (NCT02571036) BLU-285 (NCT02508532)
*BRAF*	BRAF V600E	Imatinib resistant	BRAF Inhibitors
*NF1*	NA	Imatinib resistant	MEK inhibitor trial (selumetinib) (NCT03109301)
*SDHA, B, C, D*	NA	Imatinib resistant	Sunitinib Regorafenib
*SDHCme*	NA	Imatinib resistant	Glutaminase inhibitor trial (NCT02071862) Guadecitabine trial (SGI-110) (NCT03165721)

## Conclusion

The introduction of imatinib established a new paradigm for management of solid tumors in the era of targeted therapy. Although surgical resection remains the mainstay for cure, imatinib has demonstrated an important role in the neo/adjuvant setting. Currently, three drugs are available for advanced or metastatic GISTs, however, no further standard options left if patients fail to respond to regorafenib. The need to conquer drug resistance and develop new targeted agents motivates further basic research and clinical studies. Our recent published article summarized the clinical characteristics and treatment options according to genotype of GIST (84). Intensive research of molecular pathways and new knowledge of the pathophysiology of GIST will help us to guide the personalized treatment and development of new agents.

## Author Contributions

LM and WD: drafting of work, analysis and interpretation of trials and literature, drafting of manuscript, and manuscript review. MI and MM: interpretation of trials and literature, drafting of manuscript, and manuscript review. SB: design, analysis and interpretation of trials and literature, drafting of manuscript, and manuscript review.

## Conflict of Interest Statement

The authors declare that the research was conducted in the absence of any commercial or financial relationships that could be construed as a potential conflict of interest.
